# Bioinformatics analysis and characterization of a secretory cystatin from *Thelohanellus kitauei*

**DOI:** 10.1186/s13568-020-01052-0

**Published:** 2020-06-23

**Authors:** Fengli Zhang, Yalin Yang, Chenchen Gao, Yuanyuan Yao, Rui Xia, Juan Hu, Chao Ran, Zhen Zhang, Zhigang Zhou

**Affiliations:** 1 Sino-Norway Fish Gastrointestinal Microbiota Joint Lab, Feed Research Institute, Chinese Academy of Agricultural Sciences, Beijing, 100081 China; 2 Key Laboratory for Feed Biotechnology of the Ministry of Agriculture, Feed Research Institute, Chinese Academy of Agricultural Sciences, Beijing, 100081 China

**Keywords:** TK-cystatin, *Thelohanellus kitauei*, Bioinformatics analysis, Protease inhibitor, Inhibitory activity, Molecular docking

## Abstract

*Thelohanellus kitauei*, is a member of obligate parasitic myxozoans, which causes intestinal giant-cystic disease of common carp (*Cyprinus carpio*) and has resulted in significant economic losses in carp farms. Cystatin secreted by parasites can regulate the immune response of host to facilitate parasite’s survival. In this study, the secretory TK-cystatin gene, encoding a protein of 120 amino acid residues (13.65 kDa), was cloned from *T. kitauei* genome. Phylogenetic analysis showed that TK-cystatin gene is closely related to the cystatin-A from *Hydra vulgaris*. Multiple sequence alignment revealed that TK-cystatin had three conserved motifs: N-terminal G^19^G^20^, Q^73^VVAG^77^, and C-terminal L^102^P^103^. Molecular docking between TK-cystatin and three cysteine proteases showed a lower binding energy (− 13 KJ/mol) with cathepsin L whereas a higher binding energy (− 8.6 KJ/mol) with cathepsin B. TK-cystatin gene was expressed in *Escherichia coli*. Activity assays revealed that TK-cystatin has stronger inhibitory activity on endopeptidases (papain and cathepsin L) and weaker inhibitory activity on exopeptidase (cathepsin B). TK-cystatin was stable under the condition of acidity or alkalinity or below 57 °C. This study laid a foundation for the design and development of the anti-*T. kitauei* vaccine in carp culture in the future.

## Introduction

Myxozoans, a group of obligate parasitic metazoans that are important for pathogenic effects on freshwater and marine fish, were reported in various countries of the world (Zhao et al. 2016). A number of myxozoans including *Thelohanellus kitauei* preferentially infects the intestine of host, and causes intestinal giant-cystic disease of farmed carp (Gómez et al. 2013). The *T. kitauei* parasites generally inhabit the host gut submucosa and mucosa layer of the intestines, and form cysts through continuous proliferation, which seriously damage the intestinal structure of the cultured carp, resulting malnutrition and the gradual weight loss and high mortality (Shin et al. 2011; Ye et al. 2017). *Cyprinus carpio* accounts for the largest proportion of all farmed freshwater fish and has a high production in China. However, approximately 20% of farmed carps were killed by the disease caused by *T. kitauei* in 2010, directly causing an economic loss of about 50 million dollars (Yang et al. 2014). However, due to its complicated life cycle (between fish and annelids), the infection model of *T. kitauei* was very difficult to establish in laboratory culture conditions. Therefore, the current research on *T. kitauei* was just focused on morphological research, pathological diagnosis and phylogenetic position (Eszterbauer et al. 2006; Liu et al. 2014). Our group firstly sequenced the genome and transcriptome of *T. kitauei* and found that abundant secretory proteases and protease inhibitors may be related to nutrient digestion and immune evasion of parasites (Yang et al. 2014). Research on the characterization of these proteases and protease inhibitors would pave the way for the parasite control.

Cystatin, known as cysteine protease inhibitor, was able to regulate many biological processes, and could suppress the host’s immune response to facilitate pathogen’s survival. Sialostatin, a cystatin from *hard tick*, could significantly inhibit the maturation of dendritic cells and proliferation of Ag-specific T cell (Sá-Nunes et al. 2009). OmC2 derived from *soft* *tick* could suppress the host’s adaptive immune response by reducing inflammatory cytokines and proliferation of antigen presenting cell (Salát et al. 2010). Onchocystatin could not only significantly induce the production of anti-inflammatory cytokine IL-10, but could also inhibit the synthesis of proinflammatory Th1-type cytokines and the differentiation and proliferation of regulatory Th1 cells, there by reducing the host’s immune killing effect on parasites (Schönemeyer et al. 2001). Wang et al. (2017) studied the immuno-regulatory effects of cystatin from *Haemonchus contortus* on goat monocytes and found that it could significantly inhibit the activity of goat monocytes and reduce production of the proinflammatory cytokines TNF-α, IL-1β, while significantly increasing secretion of IL-10. At the same time, cysteine protease inhibitors affected the connection between antigenic peptides and major histocompatibility complex class II (MHC-II) in the host by inhibiting the corresponding protease, which prevented the host from presenting the parasite antigen (Turk et al. 2001; Vray et al. 2002; Wang et al. 2017). Whether the structural features of a secretory cystatin (named as TK-cystatin, GenBank accession number: KII69890.1) in *T. kitauei* with high-expression level in the myxospore stage were similar to these inhibitors, and whether it could inhibit host cysteine proteases and plays similar roles?

In this study, the TK-cystatin gene was cloned from *T. kitauei* genome. Primary structure, the evolutionary relationship, conserved regions/domains, 3D structure and moculear docking of TK-cystatin were investigated by comprehensive bioinformatics analysis. TK-cystatin gene was expressed in *Escherichia coli* and its inhibitory activity and stability was characterized. This study would provide the basis for parasite-host interaction mechanism and practical applications in immunotherapy.

## Materials and methods

### Collection of *T. kitauei* from infected *C. carpio*

*T. kitauei*-infected *C. carpio* samples were collected from Wuqing, Tianjin, China. The infected *C. carpio* with the parasite had no special clinical sign beside swelling belly and it’s intestine contained multiple cysts. Extraction of the parasites was applied by previous method (Yang et al. 2014). Purified parasites were further filtrated through 40 mm nylon meshes and washed with sterile distilled water, then determined by light microscopy or stored at − 80 °C until to use. The protocol of extraction of genomic DNA was followed by previous method (Yap and Thompson. 1987). The identified 18S rDNA of the parasites (GenBank accession number: HQ115585) was amplified using specific primers (Forward primer: 5′-ATGTTGTGCTTGGTGACATTCATATTTTTTG-3′; Reverse primer: 5′-TCAATATCCCGACACGCCACC-3′). The PCR product was confirmed by DNA sequencing.

### Cloning of TK-cystatin gene

Total RNA of purified *T. kitauei* was extracted using Trizol RNA extraction reagent, according to Jaroenlak’s method (Jaroenlak et al. 2018). The extraction used as template RNA in reverse transcription reactions to produce cDNA using an oligo-dT primer, which subsequently used as the template for cloning the TK-cystatin gene. According to our early analysis of *T. kitauei* genome, the full-length of TK-cystatin gene (GenBank accession number: KII69890.1) was amplified by the specific primer pairs (Forward primer: 5′-ATGTTGAAGGCGGCTGTC-3′; Reverse primer: 5′-TCATTGCGGATGTTTTGTATCA-3′). PCR was performed with the following parameters: initiation denaturation at 94 °C for 3 min, followed by 30 cycles of 94 °C for 30 s, 53 °C for 30 s, and 72 °C for 1 min, and a final extension at 72 °C for 2 min. The PCR products were confirmed by DNA sequencing.

### Characterization and phylogenetic analysis of TK-cystatin

Bioinformatic analysis of the TK-cystatin was performed. The ExPASy Online tool (https://web.expasy.org/protparam/) was used to predict the theoretical isoelectric point (*PI*), the molecular mass, and stability coefficient. NetOGlyc 4.0 and NetNGlyc 1.0 (Steentoft et al. 2013; Gupta et al. 2004) were used to predict potential *O*-glycosylation site and *N*-glycosylation site. PROSITE (https://prosite.expasy.org/cgi-bin/prosite/ScanView.cgi?scanfile=323446234217.scan.gz) was used to predict potential phosphorylation site (Sigrist et al. 2012).

For the phylogenetic analysis, a phylogenetic tree of TK-cystatin was constructed by using neighbor-joining (NJ) method with MEGA 7.0 (Saitou and Nei 1987; Kumar et al. 2016). The evolutionary distances as described by Nei et al. (2000) were computed using the p-distance method. Test of the reality of phylogeny tree was used bootstrap method with 500 bootstrap replicates (Felsenstein 1985).

### The 3D structure and molecular docking of TK-cystatin

The homology search of TK-cystatin protein was carried out by BLASTP, and the top seven hits of similar sequences from other species, such as cystatin-A from *Hydra vulgaris* (XP_012565994), cystatin-B from *Oreochromis niloticus* (XP_003439236), cystatin-A from *Aotus nancymaae* (XP_021529657), cystatin-A from *Opisthocomus hoazin* (XP_009930686), cystatin-B from *Peromyscus maniculatus bairdii* (XP_006989515), cystatin-A from *Pelodiscus sinensis* (XP_006124242), and cystatin-A from *Chrysemys picta bellii* (XP_023966898) were selected for multiple sequence alignment using ClusterW and Espritt 3.0.

Secondary and tertiary structures of TK-cystatin were predicted using Phyre2 (Kelley et al. 2015). Model quality of predicted 3D structure was evaluated by a Ramachandran plot using Procheck analysis tools (https://servicesn.mbi.ucla.edu/PROCHECK/) (Laskowski et al. 1993). Visualization and optimization of graphics of 3D structure was executed by the PyMOL Molecular Graphics System, Version 2.0 (DeLano 2002).

To study the inhibitory mechanism of TK-cystatin against three cysteine proteases, molecular docking analysis was performed using ZDOCK 3.0.2 (http://zdock.umassmed.edu/) (Pierce et al. 2014). Three cysteine proteases obtained from PDB database: papain (PDB ID: 1ppp), cathepsin L (PDB ID: 2NDQ), cathepsin B (PDB ID: 1GMY) were selected to dock with TK-cystatin. The structure of the predicted complex were analyzed by PDBepisa online website (https://www.ebi.ac.uk/pdbe/pisa/), and the main interaction bonds were visualized in the PyMOL Molecular Graphics System, Version 2.0 (DeLano 2002).

### Heterogeneous expression and purification of TK-cystatin protein

The TK-cystatin gene was inserted into pET28a expression vector through homologous recombination, and His-tag was introduced to the c-terminus of the TK-cystatin gene for purification. Two primer pairs, pET28aF (5′-AGAAGGAGATATACCATGTTGAAGGCGGCTGTCTTTC-3′)/pET28aR (5′-TCAGTGGTGGTGGTGGTGGTGTTGCGGATGTTTTGTATCAAG-3′) and TK-cystatinF (5′-CACCACCACCACCACCACTG-3′)/TK-cystatinR (5′-CATGGTATATCTCCTTCTTAAAGTTAAA-3′) were used for amplifying the vector fragment and the target gene fragment, respectively. The PCR-amplified DNA fragments were purified and assembled into recombinant pET28a_TK-cystatin plasmid by NEB/NEBuilder^®^ HiFi DNA Assembly Master Mix (New England Biolabs). The recombination product then was transformed into *E. coli* BL21 (DE3) and positive clones were determined by DNA sequencing using T7 universal primers. A single positive clone from overnight culture was transferred to 200 mL LB medium with kanamycin (100 μg/mL) according to the ratio of 1 : 100. When the OD_600_ = 0.6-0.8, induced with various concentrations of IPTG (isopropyl β-d-1-thiogalactopyranoside) at 16 °C for overnight. The culture was centrifuged at 12,000×*g* at 4 °C for 10 min, the bacterial cells were harvested. TK-cystatin protein was purified from recombinant cells using Ni^2+^-NTA affinity method as described by Huo et al. (2016), and was dissolved at 50 mmol/L NH_4_HCO_3_ (pH = 7.5) based on previous research (Keilova and Tomášek 1974). The purified protein was analyzed by 12% SDS-PAGE and identified using MALDI-TOF–MS.

### Inhibitory activity and stability of TK-cystatin

To investigate the potential immune inhibitory function of TK-cystatin, the inhibitory activity of recombinant TK-cystatin protein was measured against three physiologically relevant peptidases including endopeptidases (papain from Carica papaya and cathepsin L from human) and exopeptidase (cathepsin B from human). All the three proteases were purchased from Sigma-Aldrich (Chen et al. 2017; Coronado et al. 2017). Fluorescent short peptide substrates from Sigma-Aldrich(Z-Phe-Arg-AMC for papain and cathepsin L, Z-Arg-Arg-AMC for cathepsin B) were diluted in DMSO for 1 mmol/L as stock concentration. The experiment was performed as previously described with some midifications (Coronado et al. 2017; Sun et al. 2013). Cysteine proteases (0.15 μmol/L for papain, 0.026 μmol/L for cathepsin L, 0.026 μmol/L for cathepsin B) in the incubation buffer (100 mmol/L sodium acetate, 100 mmol/L sodium chloride, 1 mmol/L EDTA, 1 mg/mL l-cysteine hydrochloride, 0.005% tritonx-100, pH  5.5) were activated by pre-incubation in 96 well black microplate at 37 °C for 30 min. After that, another two reactants, 10 μmol/L substrates and an increasing-concentration gradient TK-cystatin, were added to each well of this microplate to a final volume of 200 μL. Subsequently, fluorescent products were measured with excitation at 360 nm and emission at 460 nm using fluorescence spectrometer (Bio Tek) every 30 s for 10-min reaction. Each reaction was performed in triplicate.

In order to further study the properties of TK-cystatin, the effects of temperature and pH on it were measured as descripted previously (Tzeng et al. 2002). The thermal stability of TK-cystatin was assessed by assay of TK-cystatin inhibitory activity against papain with the fluorescent substrate Z-Phe-Arg-AMC after preincubating at 25, 37, 47, 57, and 67 °C for 1 h respectively. The stability of TK-cystatin at different pH values was determined by preincubating TK-cystatin in solutions with pH at 3.0 to 11.0 for 1 h, followed by assay of TK-cystatin inhibitory activity against papain under standard conditions as described above. The buffers used were disodium bicarbonate-citrate buffer (pH 3.0 to 8.0) and sodium carbonate-sodium bicarbonate buffer (pH 9.0 to 11.0).

### Statistical analysis

The activity of TK-cystatin protein was expressed as half maximal inhibitory concentration (IC_50_). It based on real-time mean velocities and determined by nonlinear regression analysis. All statistical analyses were performed in GraphPad Prism Version 6 software (GraphPad Software Inc. San Diego, CA, USA).

## Results

### Extraction and identification of *T. kitauei*

**Fig. 1:**
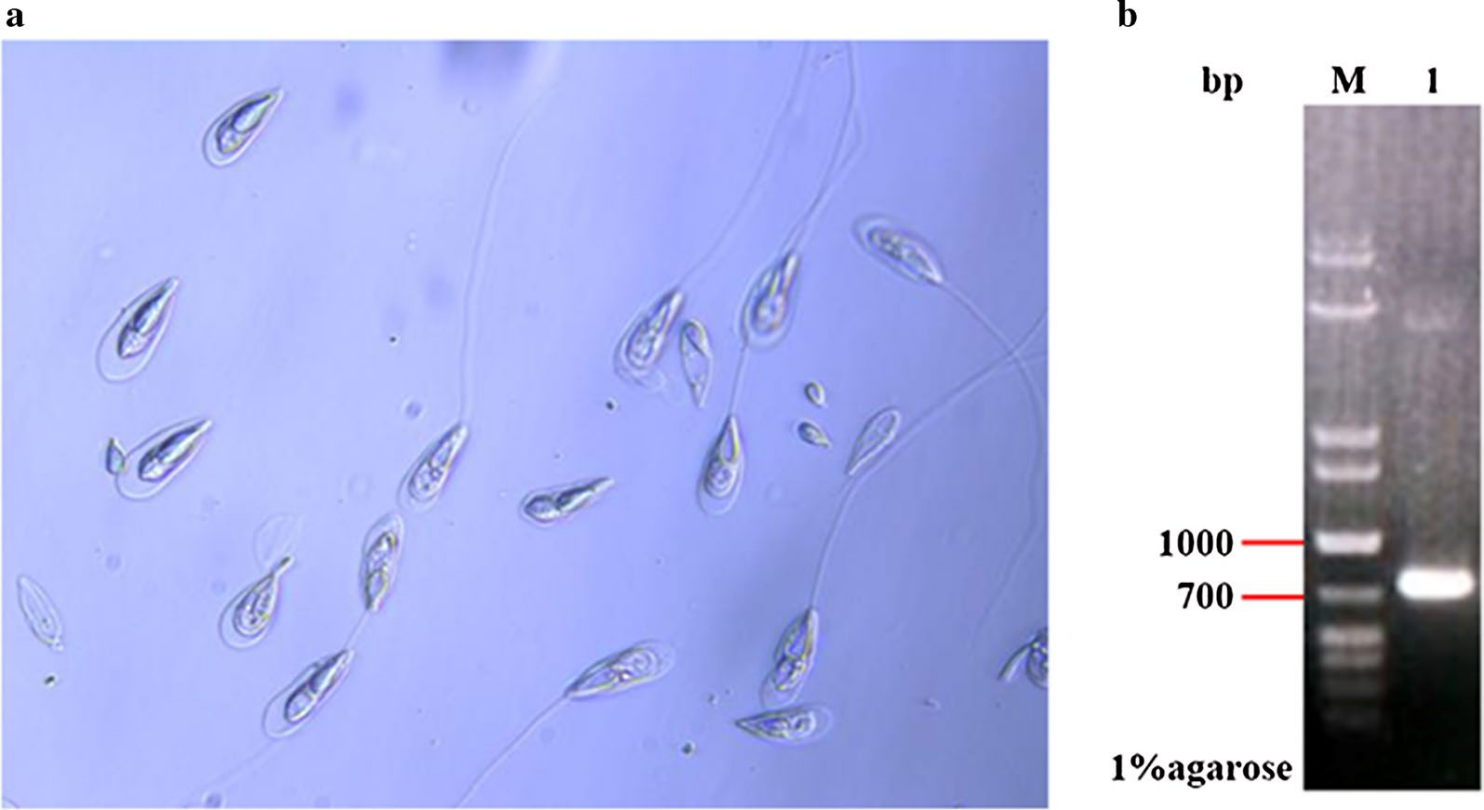
Identification of *T. kitauei*. **a** Light micrograph of parasite spores stained with 1% crystal violet solution (40X). **b** 18S rDNA PCR amplification product of *T. kitauei*. Line M: DNA marker (MD113, TIANGEN); Line 1: 18S rDNA

Dissection of infected *C. carpio* revealed that *T. kitauei* was mainly located in the cysts on the surface of the host intestine. Light microscopy revealed that these spores were pyriform in shape having a pointed front and blunt rear end, and polar filaments were either coiled spirally in the polar capsules or extruded to parasites’ surface (Fig. 1a). The size of 18S rDNA product of the isolated parasites was nearly 1000 bp in the agarose gel electrophoresis, which was consistent with its theoretical size (Fig. 1b), and it had 99% identity to that of the *T. kitauei* (GenBank accession number: HQ115585). Combined with the morphological observations, the parasite was identified as *T. kitauei.*

### TK-cystatin sequence bioinformation analysis and its phylogeny

**Fig. 2:**
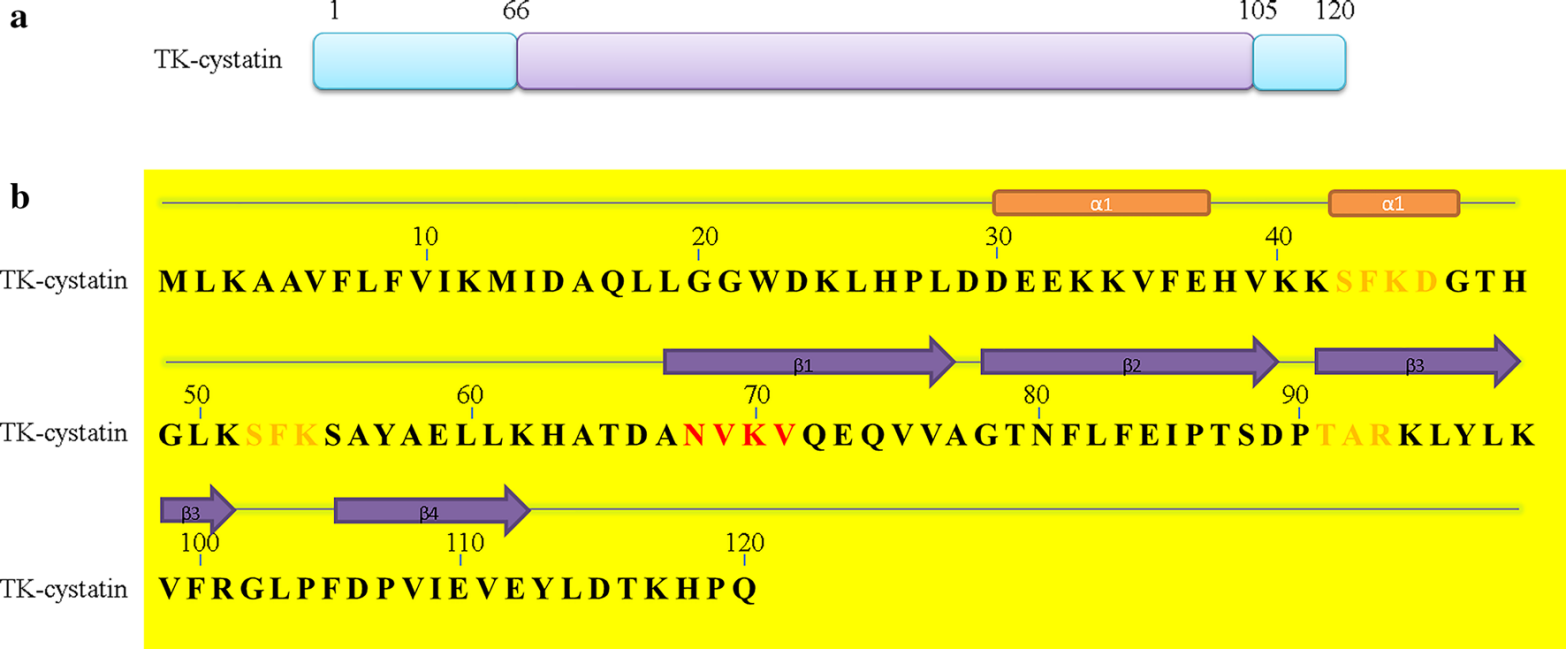
Molecular characteristics of the amino acid sequence of TK-cystatin. **a** Domain of TK-cystatin, schematic view of the position of domains in the amino acid sequence (Lavender indicated the position of the cystatin domain in the sequence). **b** The secondary structure was marked in the amino acid sequence (Red and orange represented *N*-glycosylation and phosphorylation sites; orange rectangular for α-helices, deep purple arrow for β-strand)

The 363 bp full-length TK-cystatin gene encodes 120 amino acid residues with an estimated molecular mass of 13.65 kDa and a theoretical *p*I of 6.97 predicted by the ProtParam (Fig. 2a). The low computed instability index (II) of TK-cystatin (15.82) indicated that the protein was stable. One potential N-glycosylation site (N^68^VKV^71^), one Casein kinase II phosphorylation site (S^42^FKD^45^) and three protein kinase C phosphorylation site (S^42^FK^44^、S^52^FK ^54^、T^91^AR^93^) in TK-cystatin were predicted by NetNGlyc 1.0 and PROSITE (Fig. 2b). It’s predicted that secondary structure consisted of one α-helices and four β-strand (Fig. 2b).

**Fig. 3:**
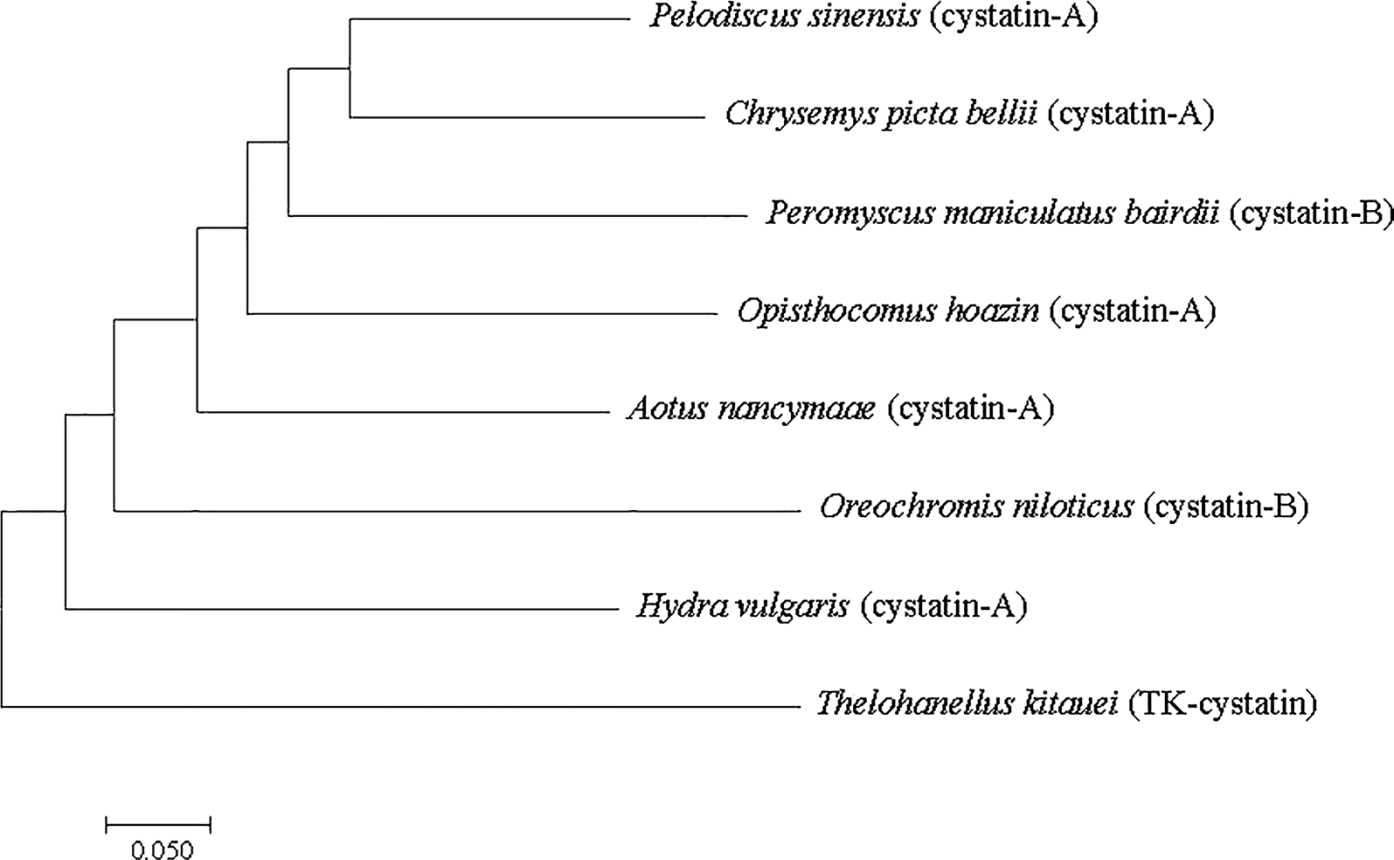
Phylogenetic tree of TK-cystatin and other species homologous cystatins. Phylogenetic tree was constructed by MEGA 7.0 using the Neighbor-Joining algorithm. Bootstrap values from 500 replicates were given on the nodes

**Fig. 4:**
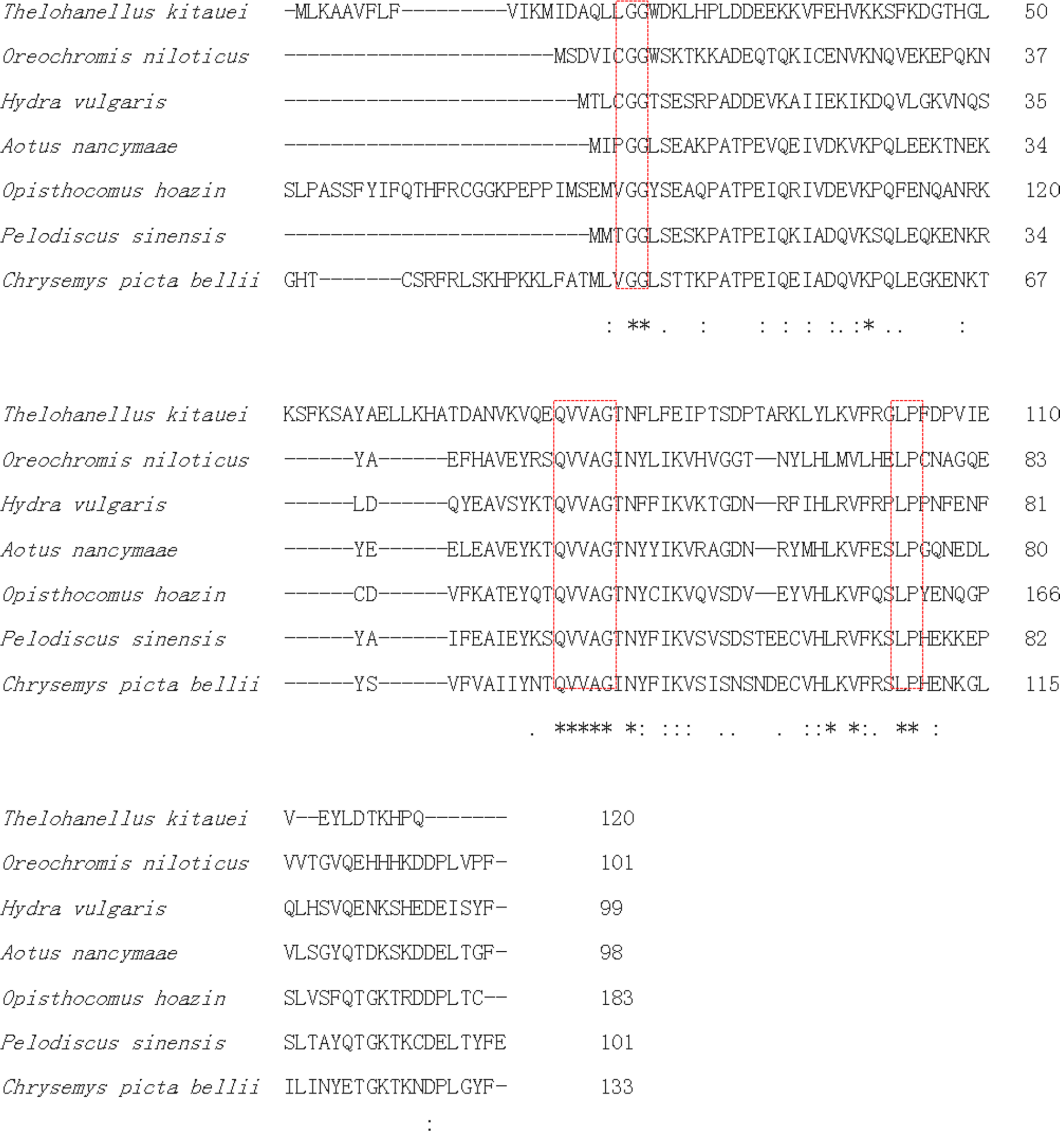
Multiple sequence alignment of TK-cystatin with other cystatins from different species. The analysis was performed by ClustalW. The numbers on the right represented the positions of the amino acids of the different sequences. The asterisk in the bottom row represented consistent sequence in different sequences. Three activity-related conserved motifs in cystatin-type protein were boxed in red

Phylogenetic analysis of cysteine protease inhibitors indicated that TK-cystatin was closely related to the cystatin-A of *H. vulgaris* cnidarian and cystatin-B of *Oreochromis niloticusi* (Fig. 3). Alignment with other cystatins indicated that TK-cystatin contained three conserved cystatin motifs (N-terminal G^19^G^20^, Q^73^VVAG^77^motif and C-terminal L^102^P^103^) critical to the biological function of cystatins (Fig. 4).

## 3D structure of TK-cystatin and molecular docking

**Fig. 5:**
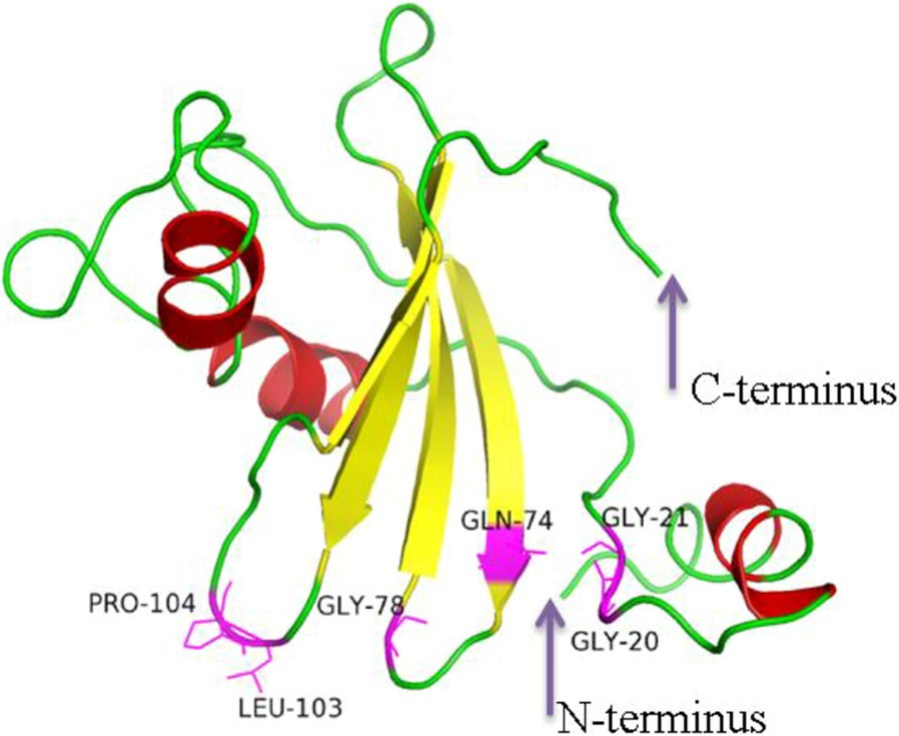
Three-dimensional structure of TK-cystatin. Its 3D structure showed the typical wedge-shaped structure of cysteine protease inhibitor. The deep purple arrows pointed to the N- terminus or C-terminus of TK-cystatin. The position of the conserved motif in the 3D structure was shown by a magenta stick, next to the residue names

Aided by Phyer2, the stefin A of family Cystatins from *Homo sapiens* was used as the template [PDB ID: d1nb5i, 2.4 Å resolution; with 28.6% sequence identity to TK-cystatin; (Jenko et al. 2003)] to predict the structure of TK-cystatin. The 3D structure model quality was evaluated by Procheck and visualized by PyMOL 2.0 (Fig. 5). The result showed that TK-cystatin had a typical wedge-shaped structure consisting of four anti-parallel β-sheets wrapping around a α-helix that was almost perpendicular to the β-sheet direction on one side and extending antiparallelly by 8 C-terminal residues on the other side. And this indicated that TK-cystatin had a similar structure to cystatinA. The N-terminal conserved region, the hairpin loop 1 region (between β1 and β2) and hairpin loop 2 region (between β3 and β4) together formed the wedge-shaped edge, which was complementary to protease cleavage. The N-terminal G^19^G^20^ was indispensable for the ability of the inhibitor to bind to the cysteine protease, with decreased affinity for inhibitor binding if deletion of this motif. The hairpin loop 1 containing the conserved QVVAG motif was a classic conformation of Cystatins. In TK-cystatin, the hairpin loop 2, formed by amino acid residues between two C-terminal β-sheet included conservative hydrophobic LP residues that was absence or replaced by PW in other cystatins (Cuesta-Astroz et al. 2014).

**Fig. 6:**
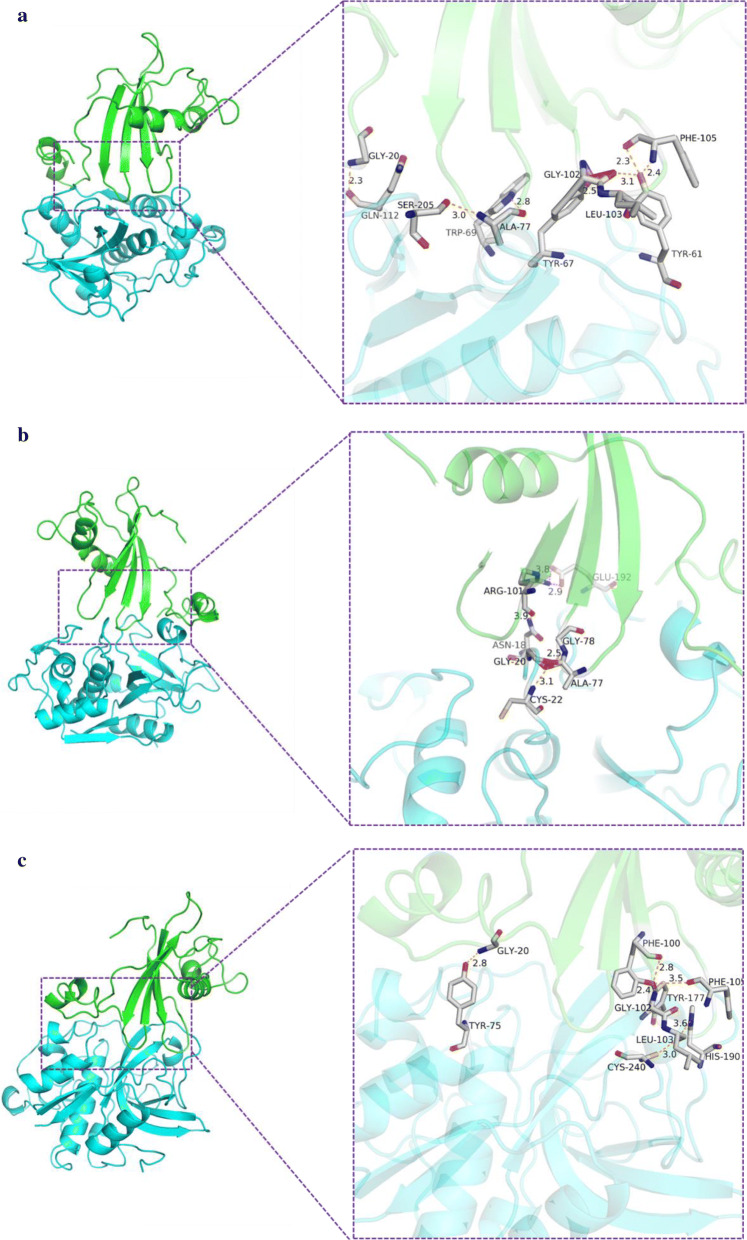
Interaction between TK-cystatin and proteases. **a** papain (PDB ID: 1ppp), **b** cathepsin L (PDB ID: 2NDQ), **c** cathepsin B (PDB ID: 1GMY). TK-cystatin was shown in green and three cysteine proteases were shown in blue. The hydrogen and salt bonds formed between the conserved site of TK-cystatin and three proteases were shown in orange and magenta, respectively

Molecular docking showed that the binding pattern between TK-cystatin and cysteine proteases was reminiscent of the shape of a “fork” that inserted into the active site of proteases from the top (Fig. 6). The loop1 of TK-cystatin was deeply inserted into shallow groove of S’ subsite of proteases and interacted with the active site of the proteases. The other two conserved domains were approximately served as a fixed function, particularly in cathepsin B, that interacted with the surface of the proteases by forming hydrogen bonds or salt bonds (Fig. 6c). In addition, some amino acid residues in the vicinity of the conserved sites also played an important role in the interaction with proteases (Fig. 6). Among three proteases docking with TK-cystatin, the interaction with cathepsin L had salt bonds in addition to hydrogen bonds (Fig. 6b). This may explain why the binding energy with cathepsin L was the lowest (− 13, − 12.4 and − 8.6 KJ/mol for cathepsin L, papain and cathepsin B, respectively). The lower binding energy between cathepsin L and TK-cystatin indicated that the complex was more stable.

### Cloning and heterogeneous expression of TK-cystatin gene

**Fig. 7:**
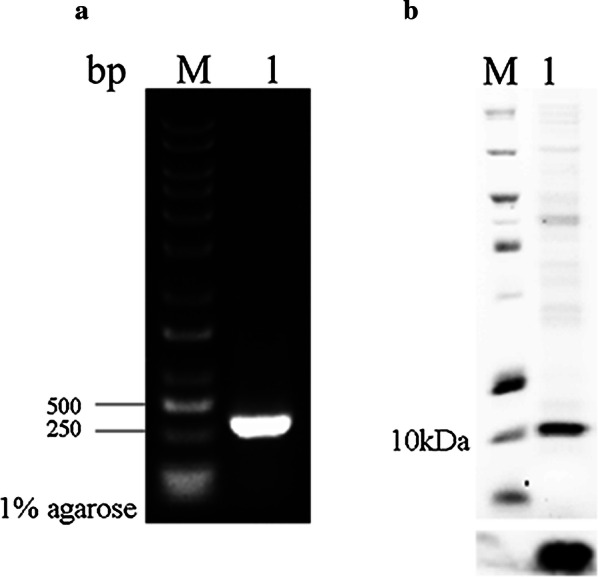
Gene cloning and purification of TK-cystatin. **a** PCR products were amplified from the genome of *T. kitauei*. Line M: DNA marker (M115, GenStar); Line 1: TK-cystatin gene. **b** Purified TK-cystatin was analyzed by 12% SDS-PAGE (up) and western blotting (down), Line M: Protein marker (26630, Thermo Scientific); Line 1: purified TK-cystatin

TK-cystatin cDNA fragments were amplified by RT-PCR from total RNA isolated from *T. kitauei*, a 363 bp band was apparently in the agarose gel electrophoresis as shown in Fig. 7a. The purified PCR products of the TK-cystatin gene was sequenced and shared 99% identity with the corresponding predicted cystatin gene in *T. kitauei* genome. TK-cystatin gene was expressed in *E. coli* BL21(DE3). After induction with 0.1 mmol/L IPTG (final concentration) at 16 °C overnight, TK-cystatin was purified from the recombinant bacteria cell lysates by Ni^2+^-NTA affinity chromatography with 200 mmol/L imidazole elution buffer. The purified protein migrated to a predicted ~ 10 kDa band in 12% SDS-PAGE, was confirmed by western blot analysis with anti-His-tag mouse monoclonal antibody (Fig. 7b) and identified by MALDI-TOF-MS analysis with 90% peptide coverage.

### Inhibitory activity and stability of TK-cystatin

**Fig. 8:**
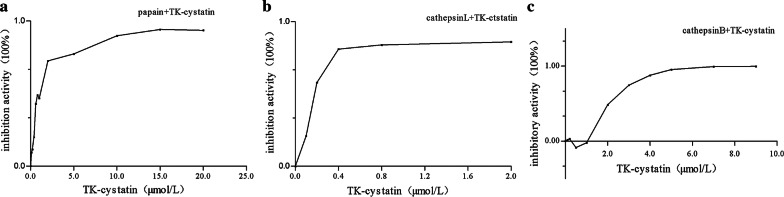
Inhibitory activity of TK-cystatin against three cysteine proteases. Inhibitory activity of TK-cystatin against papain (**a**) and cathepsin L (**b**) and cathepsin B (**c**). The activity of non-inhibited enzyme (incubated without TK-cystatin) was taken as 100% activity (Inhibitory activity: 0%). Data shown were the average of three experiments

**Fig. 9:**
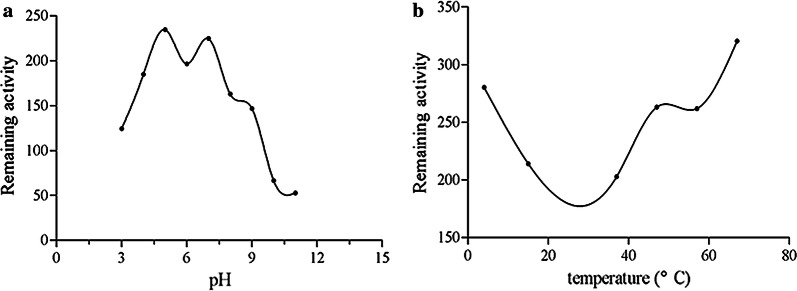
pH and thermal stability of TK-cystatin. TK-cystatin incubated at different pHs or temperatures for 1 h and assayed for residual inhibitor activity against papain at standard conditions. Data shown were the average of three experiments

Cystatin secreted by the parasite exerts immune evasion mainly by inhibiting the corresponding proteases in the host (Lecaille et al. 2002). Lysosomal cysteine proteases, especially cathepsin B, cathepsin L and cathepsin S, played an important role in immune regulation, such as could promote the binding of MHC-II molecules to antigens and affected the phagocytic activity of DC cells (Honey and Rudensky 2003; Smith et al. 2016). Cystatin could specifically inhibit papain and cathepsin protease activity through a strong binding interaction. In this study, the inhibitory activities of TK-cystatin against three standard proteases, including papain, cathepsin L and cathepsin B, were evaluated. TK-cystatin could inhibit the activities of all the three proteases in a dose-dependent manner (Fig. 8). The values of half maximal inhibitory concentration (IC_50_) for papain, cathepsin L, cathepsin B inhibition by curve fitting were 0.8 μmol/L, 0.175 μmol/L, and 2.1 μmol/L, respectively. pH and thermal stabilities of TK-cystatin were investigated. TK-cystatin had lower activity stability near pH 7.0, higher stability at alkaline or acidic conditions, with highest stability at pH 11.0 (Fig. 9a). TK-cystatin was thermally stable below 57 °C (Fig. 9b).

## Discussion

In the absence of effective drugs to treat and prevent myxozoan infections, there is an urgent need to explore potential drug targets and develop effective and safe therapeutic drugs. Cysteine protease inhibitors are a major group of protease inhibitors secreted by parasites. They can regulate both the parasite and host cysteine protease activity and play important roles in parasite infection and immune suppression of their hosts (Lecaille et al. 2002; Norbury et al. 2012). rAv17, a cystatin C from *Filarial nematodes*, can markedly suppress mitogen-induced T cell proliferation and up-regulate IL-10 product (Hartmann et al. 1997). Nippocystatin from *Nippostrongylus brasiliensis* can down-regulate the process of antigen presentation in vitro and OVA-specific IgE levels in vivo, and mice with anti-nippocystatin antibodies became resistant to infection with *N. brasiliensis* to some extent (Dainichi et al. 2001). Therefore, cystatins are often used as candidate drug targets for parasite control (Lecaille et al. 2002; Smith et al. 2016).

Cysteine protease inhibitors can be divided into three major families, namely stefins (family 1) and cystatins (family 2) and kininogens (family 3). TK-cystatin has some important features of family-2 cystatins, such as secretory, small molecular weight, rich in phosphorylation sites, and conserved inhibitory regions (motifs of G^19^G^20^, Q^74^VVAG^78^ and L^103^P^104^). TK-cystatin, however, lacks of disulfide bond, the SND/S conserved motif relating to the inhibition of legumain-like proteases (Ilgová et al. 2017), and has relatively low sequence homology to other family-2 members. Therefore, TK-cystatin is a new member of cystatins (family 2).

With the in-depth studies on the morphology, development and evolution of myxozoa, more evidences support that myxozoans are multicellular metazoans and obligately parasitic cnidarian animals (Feng et al. 2014; Zhao et al. 2016). Our molecular evolutionary analysis of TK-cystatin revealed that it was most closely related to the cystatin-A of *H. vulgaris*, also provided evidence to support this view.

TK-cysatin has the inhibitory activity on both endopeptidases (papain and cathepsin L) and exopeptidase (cathepsin B), but the inhibitory activity of it against cathepsin L is 12 times higher than that against cathepsin B. This result is consistent with the conclusion of the previous studies. Because compared to cathepsin L, cathepsin B contains an additional “occluding loop”structure in the S′ region, which usually restricts the inhibitor from entering the active site (Turk et al. 2001; Fujishima et al. 1997). MHC-II-restricted antigen presentation plays a central role in the immune response against exogenous antigens. Cathepsin L, but not cathepsin B, degrades efficiently MHC-II-associated invariant chain, which makes MHC-II peptide-binding grooves fully exposed to facilitate binding with exogenous antigen polypeptides (Manoury et al. 2001; Medd and Chain 2000; Schierack et al. 2003). The results of this experiment show that TK-cystatin has a stronger inhibitory activity on cathepsin L, indicating that the inhibitor may serve the parasite as a mean to evade the host’s immune attack.

## Data Availability

All the data were presented in the main paper.

## Abbreviations

RT-PCR: Reverse transcription-polymerase chain reaction
PCR: Polymerase chain reaction
MALDI-TOF–MS: *Matrix*-*Assisted laser Desorption/Ionization Time of Flight Mass Spectrometry*
SDS-PAGE: Sodium dodecylsulphate polyacrylamide gel electrophoresis
